# Polish translation and validation of the student satisfaction and self-confidence in learning scale among healthcare students at the Pomeranian Medical University in Szczecin: a single-center cross-sectional validation study

**DOI:** 10.3389/fmed.2026.1913838

**Published:** 2026-08-25

**Authors:** Anna Bielikowicz, Wioletta Pawlukowska, Martyna Brzosko, Elżbieta Grochans, Bogusław Machaliński, Bartłomiej Baumert

**Affiliations:** 1 Department of Hematology and Transplantology, Pomeranian Medical University, Szczecin, Poland; 2 Department of Neurology, Pomeranian Medical University, Szczecin, Poland; 3 Department of General Pathology, Pomeranian Medical University, Szczecin, Poland; 4 Department of Nursing, Pomeranian Medical University, Szczecin, Poland; 5 Medical Simulation Center, Pomeranian Medical University, Szczecin, Poland

**Keywords:** confirmatory factor analysis, cross-cultural adaptation, healthcare education, medical simulation, psychometrics, scale validation, self-confidence, student satisfaction

## Abstract

**Background:**

Simulation-based education is an established component of healthcare training, yet robust Polish-language instruments for evaluating the learner’s reaction to simulation remain scarce. The Student Satisfaction and Self-Confidence in Learning (SSCL) scale operationalizes the reaction level of educational evaluation within the NLN/Jeffries Simulation Framework. This study aimed to translate, culturally adapt, and validate the Polish version (PL-SZZ) in a single-center, convenience sample of healthcare students and, secondarily, to determine how it should be scored in practice.

**Methods:**

This cross-sectional validation study was conducted at the Pomeranian Medical University in Szczecin (data collected 2023–2025). Translation followed a five-stage forward–backward protocol with sworn translators and expert adjudication. Students who had participated in simulation classes completed the PL-SZZ anonymously. After screening for extreme observations (1.5 × interquartile range), 827 of 879 questionnaires were analysed. Confirmatory factor analysis (DWLS/WLSMV applied to polychoric correlations) compared a one-factor and a correlated two-factor model. Reliability was evaluated using Cronbach’s α, McDonald’s ω, and marginal reliability (MR); validity via standardized loadings, average variance extracted (AVE), the heterotrait–monotrait (HTMT) ratio, and added-value analysis.

**Results:**

The analytic sample comprised 827 students (86.6% female), predominantly nursing students (64.6%). The correlated two-factor model fitted better than the unidimensional model (χ^2^(101) = 401.89; CFI = 0.986, TLI = 0.989, RMSEA = 0.060, SRMR = 0.050). Standardized loadings ranged from 0.701 to 0.886 across items, and the two factors were strongly correlated (*r* = 0.860; HTMT = 0.866). Internal consistency was good (total α = ω = 0.909; subscales 0.838–0.871), whereas marginal reliability was lower (0.656–0.728); added-value coefficients were negative for both subscales (−0.071 and −0.241). Scores did not differ across simulation modalities but differed significantly between fields of study and by sex.

**Conclusion:**

In this single-center, predominantly female and nursing-dominated sample, the PL-SZZ reproduced the original correlated two-factor structure with acceptable reliability and construct validity. Because the subscales added no incremental precision, the total score is recommended for routine evaluation, with subscale scores reserved for diagnostic and curriculum-development purposes. Findings should not be generalized beyond comparable populations. Further multi-center research is needed before using the instrument for between-group or institutional comparisons.

## Background

Simulation-based education (SBE) has emerged as a transformative pedagogical methodology across medical and health professions education, reshaping how clinical competencies are developed and assessed (1, 2). By combining experiential learning with controlled practice environments, SBE bridges the gap between theoretical instruction and clinical application, allowing learners to rehearse psychomotor, cognitive, and communication skills in a safe and repeatable setting (3). Contemporary simulation encompasses a broad range of modalities, including high-fidelity manikins with sophisticated physiological responses, standardized patients, virtual reality environments, computer-based simulations, and hybrid approaches (2, 4).

The evidence supporting the effectiveness of SBE is substantial. A meta-analysis of 145 studies across multiple healthcare domains concluded that simulation represents one of the most effective pedagogical approaches for facilitating complex clinical skills acquisition (5). Documented benefits include improved psychomotor and technical performance, enhanced clinical reasoning, development of teamwork and communication competencies, and measurable gains in patient safety outcomes (3, 6, 7). Higher-fidelity simulation has been associated with superior skill performance compared with lower-fidelity approaches, with standardized mean differences of approximately 0.38 for knowledge and 0.63 for skill performance (6). Beyond technical learning, simulation consistently increases learner confidence and self-efficacy, particularly for low-frequency, high-consequence clinical scenarios (7, 8).

Simulation has also been recognized as a critical tool for patient safety, eliminating risk to real patients during skill acquisition and enabling in-situ simulation for systems-based error analysis (9). The quality of the learning climate–including psychological safety and the structure of the debriefing–is a recognized prerequisite for effective simulation learning, although neither construct is operationalized by the instrument evaluated in the present study (10, 11).

Despite these benefits, SBE implementation faces substantial challenges. Financial and infrastructural constraints, the need for skilled facilitators, incomplete evidence on transfer of learning to actual clinical practice, and heterogeneity of outcome measurement across studies all limit the field (3, 6, 12). In addition, the variability of debriefing quality and the subjective nature of learner experience underscore the importance of reliable and valid tools for evaluating the perceived educational quality of simulation (13).

In this context, monitoring learner satisfaction and self-confidence becomes essential. Universities that systematically collect structured feedback from learners gain an evidence base for continuous improvement of their simulation curricula and allocation of resources. The Student Satisfaction and Self-Confidence in Learning (SSCL) scale, developed by the National League for Nursing (NLN) and first published by Jeffries and Rizzolo in 2006, is the most widely used instrument for this purpose (14). It consists of 13 items rated on a 5-point Likert scale and yields two subscales: Satisfaction with Current Learning (5 items) and Self-Confidence in Learning (8 items). The tool has been translated and validated across numerous linguistic and cultural contexts, including Portuguese (Brazil) (15), Spanish (Chile) (16), and several other languages (17, 18), and has been used in studies evaluating virtual, high-fidelity, and standardized-patient simulation (19, 20).

Conceptually, the SSCL is anchored in the NLN/Jeffries Simulation Framework, in which learner satisfaction and self-confidence are specified as immediate, learner-level outcomes of a simulation experience, distinct from knowledge acquisition, skill performance, and transfer to practice (14, 21). In evaluation terms, the instrument therefore operationalizes the reaction level (Level 1) of Kirkpatrick’s model of educational evaluation (22), while its self-confidence dimension additionally draws on Bandura’s construct of self-efficacy–the learner’s belief in their capacity to execute the behaviors a clinical situation requires (23). This framework has two consequences that guided the present study. First, the SSCL is not a measure of competence, and its scores must be interpreted as the learner’s appraisal of an educational experience rather than as evidence of learning. Second, because satisfaction and self-efficacy are theorized as related but conceptually separable outcomes of the same experience, the empirical question of whether they can be measured separately in a given population is a genuine one, and is addressed directly below.

Internationally, the measurement properties of the SSCL have been examined repeatedly, with broadly convergent but not identical results. The Brazilian Portuguese adaptation supported the original two-dimensional structure and reported good internal consistency (15). The Spanish adaptation, conducted in 489 nursing students, found both a unidimensional and a two-dimensional solution to be acceptable, with Cronbach’s α of 0.88 for the total scale (24). Validation in United States pre-licensure learners supported the two-factor solution (18), and evaluations in Chilean, Turkish, and Norwegian samples reported adequate internal consistency (16, 17, 19). Two observations recur across these studies and are directly relevant to the present work: the two subscales are consistently and strongly intercorrelated, which raises the question of essential unidimensionality, and responses show pronounced ceiling effects in satisfied volunteer samples. Neither issue has been formally examined in a Polish sample, and no previous adaptation of the instrument in any language has tested whether the subscale scores carry information beyond the total score.

In Poland, there are currently approximately 85 Medical Simulation Centers. Despite the rapid expansion of simulation-based teaching, systematic evaluation of its educational quality is uncommon, and Polish-language instruments for this purpose are scarce. To date, only Studnicka et al. have conducted a Polish validation of the SSCL, in a sample of 361 nursing students at two centers (25). Their study confirmed a two-dimensional factor structure and high internal consistency, but was limited to nursing students and used classical exploratory methods without more advanced modern psychometric approaches tailored to ordinal Likert data, and the confirmatory evidence they reported was limited.

An important gap therefore remains: a Polish version of the SSCL validated in a large, multidisciplinary sample of healthcare students using contemporary confirmatory methods appropriate for ordinal questionnaire data. To our knowledge, the present study is the first Polish adaptation of the SSCL evaluated with an estimator designed for ordinal responses and, more importantly, the first adaptation in any language to combine evidence about the latent structure of the instrument with score-level evidence–marginal reliability and an empirical added-value analysis–that speaks directly to how the instrument should be scored in everyday educational practice. This combination is the principal novel contribution of the study.

The study pursued two objectives that should be kept separate. The primary objective was to translate and culturally adapt the SSCL into Polish (PL-SZZ) and to evaluate its internal structure, reliability, and construct validity among healthcare students at a single Polish medical university. The secondary objective was measurement-focused: to establish how PL-SZZ scores should be reported–as a single total score, as two subscale scores, or both–and to describe how scores are distributed across the disciplines represented in the sample. Evaluating the usefulness of the instrument for judging or comparing simulation programmes was explicitly outside the scope of this study, because such use would require external criteria of educational effectiveness and evidence of measurement invariance that the present cross-sectional, single-center design cannot supply.

## Materials and methods

### Study design and setting

This cross-sectional validation study was conducted between October 2022 and May 2025 at the Medical Simulation Center of the Pomeranian Medical University in Szczecin, Poland–a single academic center. The period given refers to the study as a whole: it opened in October 2022 with preparation of the protocol, completion of the translation procedure, and submission of the documentation to the Bioethics Committee. Administration of the questionnaire began later, at the end of the 2022/2023 academic year, so that all questionnaires were in fact collected during the calendar years 2023–2025. Students enrolled in undergraduate healthcare programs at the Faculty of Medicine and Dentistry and the Faculty of Health Sciences, who had participated in simulation-based classes using low-fidelity simulation, high-fidelity simulation, or standardized/simulated patient encounters, were eligible to participate.

### Participants and recruitment

Students were recruited at the end of their simulation-based class blocks across academic years 2022/2023 to 2024/2025. Inclusion criteria were: (i) enrolment in a healthcare program at the Pomeranian Medical University in Szczecin (Medicine, Nursing, Obstetrics/Midwifery, or Emergency Medical Services); (ii) completion of at least one simulation-based class at the Medical Simulation Center prior to the survey; and (iii) informed willingness to participate. Participation was voluntary and anonymous; students were informed that completion and submission of the questionnaire implied consent to participate. No personal identifiers were collected. Students who did not attend simulation classes or who submitted incomplete questionnaires were excluded.

Recruitment followed a consecutive, non-probability (convenience) strategy. During the study period, every class group finishing a simulation block at the Medical Simulation Center was invited to take part, in person and at the end of the final session of the block, by a teacher who was not a member of the research team. No group was selected or excluded on the basis of discipline, year of study, or simulation modality. However, the opportunity to invite a group depended entirely on the teaching schedule of the Center, so the distribution of disciplines in the resulting sample reflects the volume of simulation teaching delivered to each programme rather than the relative size of the corresponding student populations. This is the principal reason for the uneven representation of the four fields of study reported below, and it is a design feature that constrains the generalizability of the findings.

The number of questionnaires physically distributed was not recorded, because forms were handed to whole class groups and returned anonymously to a sealed collection box. A precise institutional response rate can therefore not be computed. Teaching staff reported that refusals were rare and that the great majority of students present completed the form, which is consistent with the volume of questionnaires returned.

A total of 879 questionnaires were initially collected. Questionnaires that were not completed in full on all 13 SSCL items were not entered into the analytic database at the data-entry stage. Consequently, the dataset of 879 records contained no item-level missing values. Prior to the psychometric analyses, the dataset was screened for extreme observations. Outliers were identified separately for the total score and for each of the two subscale scores using the 1.5 × interquartile range (IQR) criterion, applied *a priori* and uniformly to all records. Because the item distributions are strongly negatively skewed, this criterion isolates implausibly low or internally inconsistent response profiles–including uniform, straight-line patterns at the lower end of the response scale–and therefore serves simultaneously as the screening rule for response-pattern artifacts. Cases identified as outliers on at least one of these distributions were excluded from further analyses. This procedure removed 52 of the 879 records (5.9%); the final analytic sample comprised 827 responses (*N* = 827). This sample size substantially exceeds commonly recommended thresholds for confirmatory factor analysis of a 13-item instrument (≥10–20 respondents per item and/or ≥300 respondents) (26).

### Instrument and translation procedure

The Student Satisfaction and Self-Confidence in Learning (SSCL) scale is a 13-item instrument developed by the National League for Nursing (NLN) and first published by Jeffries and Rizzolo, (14). It comprises two subscales: Satisfaction with Current Learning (5 items) and Self-Confidence in Learning (8 items). Each item is rated on a 5-point Likert scale (1 = strongly disagree, 5 = strongly agree). Written permission to translate and use the scale was obtained from the NLN in November 2021.

The translation process followed internationally recommended cross-cultural adaptation procedures (27), operationalized in five sequential stages and additionally informed by the ISPOR principles of good practice for translation and cultural adaptation (28).

Stage 1 – forward translation. Three independent forward translations into Polish were produced by three sworn translators working from the original English instrument, one of whom was briefed on the construct being measured and two of whom were not.

Stage 2 – expert synthesis and content review. Three academic teachers of the Pomeranian Medical University, all experienced in simulation-based education and independent of one another, rated every candidate item version on a 1–3 scale for semantic clarity and for relevance to the Polish educational context. The highest-rated versions were synthesized into a single reconciled Polish draft. Residual disagreements were resolved by discussion until consensus was reached.

Stage 3 – back-translation. The reconciled draft was back-translated into English by an independent bilingual translator who had not seen the original instrument, and the back-translation was compared item by item with the source by the research team in order to identify semantic discrepancies.

Stage 4 – harmonization and cultural adjustment. Discrepancies identified at Stage 3 were resolved in a consensus meeting of the research team and the expert panel. Two items required cultural rather than purely linguistic adjustment. Items 10 and 13 refer to the instructor’s responsibility for the learning process; a literal Polish rendering implied a degree of teacher accountability for the learning outcome that does not correspond to the more student-directed framing of Polish higher-education regulations, and the wording was therefore adjusted to express the instructor’s role in supporting the student’s learning while preserving the semantic content of the original. In addition, the term denoting the learning setting was harmonized with the expression used in Polish simulation centers (“zajęcia symulacyjne”), and the response anchors were adapted to the conventional Polish agreement wording. No item was added, removed, or split.

Stage 5 – finalization. A translation protocol documenting each stage, together with the decisions taken and their justification, was prepared and signed by all individuals involved.

The final Polish version was titled “Satysfakcja studenta i pewność siebie w nauce (PL-SZZ).” The Polish version is provided in Supplementary File 1, the English version in Supplementary File 2.

Two boundaries of this procedure should be stated explicitly. Expert agreement was quantified only as the ordinal 1–3 ratings described in Stage 2; formal item-level and scale-level content validity indices (I-CVI and S-CVI) in the sense of Polit and Beck (29) were not computed. Furthermore, no cognitive-debriefing round with students preceded the main data collection. The evidence for the content validity of the Polish version therefore rests on structured expert review and back-translation rather than on quantified content-validity indices.

### Data collection

The PL-SZZ was administered in paper format at the end of simulation-based class blocks. Alongside the 13 items, participants provided demographic and educational information: sex, faculty, field of study, year of studies, and type of simulation experienced (high fidelity, low fidelity, or standardized/simulated patient), the last of these being recorded by the student with reference to the block just completed. Students completed the questionnaire in class under supervision of a teacher not involved in the research team, were informed that the survey was anonymous and voluntary, and were told that participation or non-participation would not affect their academic evaluation. Completed questionnaires were deposited into a sealed collection box to further protect anonymity.

### Ethics

The study was conducted in accordance with the Declaration of Helsinki. By decision No. KB.006.20.2023 of the Bioethics Committee of the Pomeranian Medical University in Szczecin, the need for formal ethics approval and written informed consent was waived, given the anonymous and non-interventional nature of the study. Voluntary participation and anonymity were ensured throughout the data collection process.

### Statistical analysis

Analyses were performed in JASP (version 0.98.1, based on the R statistical environment). Statistical significance was set at α = 0.05 (two-sided). Prior to confirmatory factor analysis, data were screened for missing values, response patterns, and suitability for factor analysis. Bartlett’s test of sphericity and the Kaiser–Meyer–Olkin (KMO) measure of sampling adequacy was computed. Because SSCL items are ordinal Likert-type variables, departures from multivariate normality were expected and were formally assessed using Shapiro–Wilk and Mardia tests; these results guided the choice of a robust estimator.

Descriptive statistics (mean, standard deviation, median, median absolute deviation, minimum, maximum, skewness, and excess kurtosis) were computed for each item and for the two subscales and the total score.

Confirmatory factor analysis (CFA) was performed using the weighted least squares mean- and variance-adjusted (WLSMV) estimator, which is specifically designed for ordinal data and is robust to violations of multivariate normality commonly observed with Likert-scale responses (30, 31). Analysis was conducted with use of the SEM module in lavaan emulation mode. Two competing models were specified and compared: (1) a one-factor model with all 13 items loading on a single latent construct and (2) a correlated two-factor model with items loading on their theoretically assigned factors (Satisfaction and Self-confidence). A second-order model was not retained. Although such a model can be fitted, with only two first-order factors it is statistically equivalent to the correlated two-factor solution and adds no testable structure, and there is no theoretical argument in the NLN/Jeffries Framework that requires a superordinate factor. It was therefore removed from the analysis plan in the interest of parsimony and clarity.

Model fit was evaluated using multiple indices: the chi-square test, the normed chi-square (χ^2^/df), the Comparative Fit Index (CFI), the Tucker–Lewis Index (TLI), the Root Mean Square Error of Approximation (RMSEA) with its 90% confidence interval, and the Standardized Root Mean Square Residual (SRMR). In addition to classical cutoffs (CFI/TLI ≥ 0.95, RMSEA ≤ 0.06, SRMR ≤ 0.08) (32), we applied two complementary modern approaches: the equivalence-testing approach of Yuan et al. (33), which derives condition-specific thresholds for CFI and RMSEA, and the tailored-cutoff approach of Groskurth et al. (34), which produces cutoffs adjusted to data characteristics based on the regression formulae.

Reliability was evaluated using three complementary indices: Cronbach’s α, McDonald’s ω (computed from CFA loadings), and the marginal reliability coefficient (ρ^2^), which quantifies the precision of factor score estimates. Construct validity was examined through standardized factor loadings and average variance extracted (AVE). Discriminant validity between the factors was assessed using the Heterotrait–Monotrait ratio (HTMT) (35), evaluated against both the conservative threshold of 0.85 and the liberal threshold of 0.90 proposed for conceptually similar constructs. Because a key applied question is whether subscale scores provide information beyond the total score, an added-value analysis was performed following Ferrando and Lorenzo-Seva (36) with use of FACTOR program (version 12.6.8.0) (37). This analysis compares the marginal reliability of each subscale with its squared correlation with the total score. Negative added value indicates that the total score is more reliable for measuring the subscale’s intended dimension than the subscale itself.

Multi-group confirmatory factor analysis to test measurement invariance across fields of study or between women and men was not performed. The composition of the sample does not permit it: the smallest discipline group (Emergency Medical Services, *n* = 45) and the male subgroup (*n* = 111) are far below the size required for stable WLSMV estimation of a 13-item two-factor model within each group simultaneously, so any configural, metric, or scalar invariance test conducted on these data would have been severely underpowered and its results uninterpretable.

As sensitivity analyses addressing the heterogeneity of the sample, PL-SZZ total and subscale scores were compared across the three simulation modalities and across the four fields of study using the Kruskal–Wallis test, and between women and men using the Mann–Whitney U test, with rank-biserial correlation or ε^2^ reported as effect sizes. These analyses are descriptive controls for possible confounding rather than hypothesis tests of substantive interest, and–because measurement invariance was not established–the resulting differences are reported as differences in observed scores and are explicitly not interpreted as evidence of true differences on the latent dimensions.

Classical item-analysis indices, in particular corrected item–total correlations and item difficulty in the classical-test-theory sense, are not reported. With uniformly high endorsement and a median at the scale maximum for every item, indices of this family are dominated by the restricted variance of the responses and would have been uninformative and potentially misleading. The standardized factor loadings estimated from polychoric correlations, which are not attenuated in the same way, serve the equivalent function in this analysis. The absence of a classical item-discrimination analysis is nevertheless acknowledged among the limitations.

## Results

### Sample characteristics

Of the 879 initially collected questionnaires, 827 were retained for analysis after excluding observations identified as outliers using the 1.5 × interquartile range (IQR) criterion, that is, after the exclusion of 52 records (5.9% of those collected).

The final sample consisted predominantly of female students (*n* = 716, 86.6%), reflecting the demographic structure of healthcare programmes in Poland. Participants represented four fields of study: Nursing (*n* = 534, 64.6%), Obstetrics (*n* = 131, 15.8%), Medicine (*n* = 117, 14.1%), and Emergency Medical Services (*n* = 45, 5.4%). Students from all years of study were included, with the largest proportion enrolled in the second year (*n* = 466, 56.3%). Regarding simulation modality, 57.2% of participants attended high-fidelity simulation sessions, 24.7% completed low-fidelity simulation training and 18.1% participated in standardized patient scenarios (Table 1).

**TABLE 1:** Demographic and educational characteristics of study participants (*N* = 827).

Variable	Category	*n*	%
Sex	Male	111	13.4
	Female	716	86.6
Faculty	Medicine and Dentistry	117	14.1
	Health Sciences	710	85.9
Field of study	Nursing	534	64.6
	Obstetrics	131	15.8
	Medicine	117	14.2
	Emergency Medical Services	45	5.4
Year of studies	I	41	5.0
	II	466	56.3
	III	210	25.4
	IV	32	3.9
	V	26	3.1
	VI	52	6.3
Type of simulation	High fidelity	473	57.2
	Low fidelity	204	24.7
	Standardized patient	150	18.1

The composition of this sample should be kept in view when reading every result that follows. It is a convenience sample drawn from a single university, in which women constitute 86.6% of respondents and nursing students 64.6%, and in which Emergency Medical Services students (*n* = 45) and male students (*n* = 111) are present in numbers sufficient for description but not for separate psychometric modeling. Accordingly, the analyses below characterize how the PL-SZZ behaves in a population of this composition–predominantly female healthcare students, mostly in nursing, at one Polish medical university with an established simulation center–and not in Polish healthcare students in general.

To assess the potential influence of sample heterogeneity, additional comparative analyses were conducted across simulation modality, field of study, and sex. No statistically significant differences in PL-SZZ scores were observed between the three simulation modalities, which argues against confounding by simulation fidelity in the pooled analyses. In contrast, significant differences were identified across fields of study and between female and male students. Detailed results are presented in Supplementary Table 1. As set out in the section “Statistical analysis,” these differences are differences in observed scores; because measurement invariance could not be tested in this sample, they cannot be attributed with confidence to genuine differences in satisfaction or self-confidence rather than to differential item functioning between the groups, and they are reported here as a caution against between-group use of the instrument rather than as a substantive finding.

### Data screening and descriptive statistics

Bartlett’s test of sphericity was highly significant (χ^2^ = 10012.26, *p* < 0.001) and the Kaiser–Meyer–Olkin measure of sampling adequacy was good (mean MSA = 0.91), supporting the factorability of the correlation matrix. As expected for ordinal Likert data, both Shapiro–Wilk and Mardia tests indicated departures from univariate and multivariate normality (all *p* < 0.001), justifying the use of the robust WLSMV estimator in subsequent confirmatory analyses.

Item means ranged from 4.42 (item 6) to 4.80 (item 13) on the 5-point response scale, with an overall mean of 4.67 (SD = 0.38), indicating uniformly positive endorsement. All items showed negative skewness (Sk = −0.89 to −2.15) and all medians concentrated at 5.0, consistent with ceiling effects typically observed in volunteer samples in educational settings. Scale-level distributions similarly showed moderately strong negative skewness (Sk = −0.93 to −1.16). Detailed item- and scale-level descriptive statistics are provided in Supplementary Table 2.

### Confirmatory factor analysis and model fit

Two competing measurement models of the PL-SZZ were evaluated using confirmatory factor analysis (CFA): a one-factor model representing a unidimensional structure and the original correlated two-factor model comprising the Satisfaction and Self-confidence dimensions. The correlated two-factor model demonstrated consistently better fit to the data and was therefore retained as the final measurement model.

The goodness-of-fit indices obtained for both competing models are summarised in Table 2. The one-factor model showed acceptable fit (χ^2^(103) = 642.69, *p* < 0.001, χ^2^/df = 6.24; CFI = 0.975, TLI = 0.981, RMSEA = 0.080, 90% CI [0.074, 0.086], SRMR = 0.066). The correlated two-factor model demonstrated improved fit across all primary fit indices (χ^2^(101) = 401.89, *p* < 0.001, χ^2^/df = 3.98; CFI = 0.986, TLI = 0.989, RMSEA = 0.060, 90% CI [0.054, 0.066], SRMR = 0.050), thereby providing empirical support for the original two-factor structure of the SSCL. Fit indices evaluated against the condition-specific tailored cut-offs of Groskurth et al. shown sub-optimal fit, although fit indices were generally better for the two-factor model (besides CFI) (34). Equivalence-testing thresholds of Yuan et al. (33) also shown better fit of the two-factor model. Comparisons of tailored cut-offs are reported in Supplementary Table 3.

**TABLE 2:** Goodness-of-fit indices for the two competing confirmatory factor models (*N* = 827).

Fit index	One-factor model	Correlated two-factor model
χ^2^	642.69	401.89
df	103	101
χ^2^/df	6.24	3.98
CFI	0.975	0.986
TLI	0.981	0.989
RMSEA	0.080	0.060
RMSEA 90% CI	0.074–0.086	0.054–0.066
SRMR	0.066	0.050

CFI, Comparative Fit Index; TLI, Tucker–Lewis Index; RMSEA, Root Mean Square Error of Approximation; CI, confidence interval; SRMR, Standardized Root Mean Square Residual. Condition-specific tailored cutoffs [Groskurth et al. (34)] and equivalence-test cutoffs [Yuan et al. (33)] are reported in Supplementary Table 3.

All standardized factor loadings were statistically significant (*p* < 0.001). Standardized loadings ranged from 0.754 to 0.886 for the Satisfaction factor and from 0.701 to 0.871 for the Self-confidence factor. The latent factors were strongly correlated (*r* = 0.860, *p* < 0.001), indicating that satisfaction and self-confidence represent closely related dimensions of students’ experiences during clinical simulation. Full standardized factor loadings are provided in Supplementary Table 4.

Convergent validity was supported by the average variance extracted (AVE), which exceeded the recommended threshold of 0.50 (38) for both latent dimensions. AVE equalled 0.728 for the Satisfaction factor and 0.656 for the Self-confidence factor (Table 3), indicating that both constructs explained a substantial proportion of the variance in their respective indicators. The heterotrait–monotrait ratio (HTMT) between the two factors was 0.866, reflecting the close conceptual relationship between satisfaction and self-confidence. Path diagrams of both models are provided in Supplementary Figures 1, 2.

**TABLE 3:** Reliability and validity indices (*N* = 827).

Score	Cronbach’s α	McDonald’s ω	AVE
Satisfaction subscale	0.924	0.945	0.728
Self-confidence subscale	0.934	0.953	0.656
Total scale	0.955	0.977	0.644

AVE, average variance extracted. MR, marginal reliability. The identical AVE and MR values are expected for the present standardized CFA model and reflect the mathematical relationship between the two indices rather than a computational error.

### Reliability

The internal consistency of the PL-SZZ was assessed using Cronbach’s α, McDonald’s ω, and marginal reliability (MR) (Table 3). The total scale demonstrated good reliability, with α = 0.955, ω = 0.977, and MR = 0.683. At the subscale level, the Satisfaction dimension yielded α = 0.924, ω = 0.945, and MR = 0.728, whereas the Self-confidence dimension yielded α = 0.934, ω = 0.953, and MR = 0.656.

Cronbach’s α and McDonald’s ω indicated very good internal consistency for both the total score and the two subscales. Marginal reliability coefficients were lower than the corresponding internal consistency estimates, reflecting the proportion of reliable variance attributable to the latent variables in the CFA model. Nevertheless, the obtained values indicate satisfactory reliability of the latent factor scores.

The gap between the internal-consistency coefficients and the marginal reliability estimates deserves explicit comment, because it can be mistaken for an inconsistency. The two families of coefficients answer different questions. Cronbach’s α and McDonald’s ω quantify how consistently the items covary in this sample; marginal reliability quantifies how precisely an individual respondent’s position on the latent factor can be recovered from those item responses. With strongly negatively skewed ordinal items and a median at the scale maximum for every item, the great majority of respondents are located in a narrow region at the upper end of the latent continuum, where the item information function is comparatively flat. Factor scores in that region are estimated with less precision than the covariation among items alone would suggest, and the marginal reliability values of 0.66–0.73 reflect exactly this. The practical consequence is specific: these values are adequate for group-level use–comparing cohorts, monitoring a simulation center over time, evaluating a curricular change–but they are not adequate for decisions about individual students, and PL-SZZ scores should not be used for that purpose. They also indicate where instrument development should be directed, namely toward items that discriminate at the upper end of the scale.

### Added-value analysis

Table 4 presents the results of the empirical added-value analysis following Ferrando and Lorenzo-Seva (36). For both Satisfaction and Self-confidence, the proportional reduction in mean squared error (PRMSE) based on the total score exceeded the marginal reliability of the corresponding subscale factor score estimate, resulting in negative added-value coefficients (AV = −0.090 and AV = −0.187, respectively). According to the empirical added-value criterion, these findings indicate that separate subscale scores do not provide greater predictive accuracy than the total score for estimating the corresponding latent dimensions. At the same time, this result does not contradict the superior fit of the correlated two-factor CFA model. Rather, it suggests that although Satisfaction and Self-confidence represent distinguishable latent constructs, they are very closely related and the total score captures most of the information contained in the individual subscales.

**TABLE 4:** Results of the added-value analysis (*N* = 827).

Score	r	MR	PRMSE	AV
Satisfaction subscale	0.894	0.709	0.799	−0.090
Self-confidence subscale	0.947	0.710	0.897	−0.187

*r* = correlation between the subscale factor score estimate and the general factor score estimate; MR = marginal reliability (ORION) of the fully-informative prior oblique EAP factor score estimate; PRMSE, proportional reduction in mean squared error; AV, added value. Marginal reliability (MR) was obtained from the ORION coefficient estimated by FACTOR. The PRMSE from the total score was computed as the squared correlation (r^2^) between the general factor score estimate and the corresponding subscale factor score estimate. Added value (AV) was calculated as the difference between the marginal reliability of the subscale factor score estimate and the PRMSE obtained from the total score (AV = MR−PRMSE). Positive values indicate that the subscale factor score estimate is more precise than the total score for estimating the corresponding latent dimension, whereas negative values indicate that the total score is at least as precise.

It is worth being precise about what this does and does not imply. The added-value criterion concerns the accuracy with which a score estimates its own latent dimension; it does not establish that the two dimensions are conceptually identical, nor does it render the subscales meaningless as descriptive information. The practical reading is that, in a population of this composition, the total score should be the default reporting unit, while the subscale scores retain a descriptive, diagnostic role at group level. The implications of this distinction for educational practice are developed in the Discussion.

## Discussion

The present study provides the first comprehensive validation of the Polish version of the Student Satisfaction and Self-Confidence in Learning scale (PL-SZZ), using confirmatory factor analysis tailored to ordinal data. The findings support the original correlated two-factor structure proposed by Jeffries and Rizzolo (14), with the two-factor model demonstrating better fit than the unidimensional alternative. Furthermore, the scale demonstrated high internal consistency and provided evidence of satisfactory construct validity, including convergent and discriminant validity. At the same time, the added-value analysis indicated that the total score predicted the latent dimensions at least as accurately as the corresponding subscale scores. Taken together, these findings suggest that although satisfaction and self-confidence represent distinguishable but closely related constructs, reporting subscale scores may offer limited advantages over the total score when the primary objective is to estimate the underlying latent dimensions.

These findings are generally consistent with previous validation studies of the SSCL. The original correlated two-factor structure has also been supported in Portuguese (15), Spanish (16, 24), and the previous Polish adaptation by Studnicka et al. (25), suggesting that the distinction between satisfaction and self-confidence is reproducible across different educational and cultural settings. The present study extends this evidence by confirming the proposed structure in a heterogeneous sample including students of nursing, medicine, midwifery, and emergency medical services. Unlike previous validation studies, it also incorporates contemporary psychometric approaches, including confirmatory factor analysis with WLSMV estimation, marginal reliability, and empirical added-value analysis. Together, these complementary methods provide a more comprehensive evaluation of both the latent structure of the PL-SZZ and the practical implications of interpreting its total and subscale scores.

Placing the present estimates alongside international adaptations reveals both convergence and instructive divergence. The standardized loadings observed here (0.70–0.89) and the internal-consistency coefficients (α = 0.92–0.98) are of the same order as those reported for the Spanish (24), Brazilian Portuguese (15), and United States (18) versions, and the two-factor structure was supported in each of them. The inter-factor correlation obtained in the present sample (*r* = 0.86, HTMT = 0.87) is, however, at the upper end of the published range, and the Spanish validation likewise found a unidimensional solution acceptable (24). Three explanations are plausible and are not mutually exclusive. First, sample composition differs: most previous validations were conducted in nursing students alone, whereas the present sample mixes four disciplines whose simulation experiences differ in content and duration, which may increase the weight of a general, undifferentiated appraisal of “the class” relative to its specific components. Second, the ceiling effects observed here are pronounced, and restriction of range at the upper end of both dimensions inflates their apparent association. Third, the use of polychoric correlations with a DWLS/WLSMV estimator, rather than the Pearson-based maximum-likelihood approaches employed in several earlier studies, systematically yields higher loadings and higher factor correlations for skewed ordinal items, so part of the difference between our estimates and earlier ones is methodological rather than substantive. Taken together, these considerations indicate that the Polish data are broadly consistent with the international evidence base, while indicating that, in this population, the two dimensions may represent closely related facets of a single overall appraisal of the simulation experience.

A novel contribution of this work is the application of modern psychometric methods that extend beyond traditional evaluations based primarily on internal consistency coefficients and factor structure. In addition to confirmatory factor analysis, we examined marginal reliability, HTMT, and empirical added-value analysis to obtain a more comprehensive assessment of score quality and interpretation. Whereas the CFA supported the distinction between satisfaction and self-confidence as two closely related latent constructs, the added-value analysis indicated that the total score estimated these constructs at least as accurately as the corresponding subscale scores. These findings illustrate that evidence regarding latent structure and evidence regarding score interpretation address complementary rather than identical psychometric questions. Accordingly, the choice between reporting the total score and the subscale scores should be guided by the intended purpose of the assessment rather than by model fit alone. To our knowledge, this is the first application of an empirical added-value analysis to any language version of the SSCL, and it is what allows the present study to move from the question “Does the instrument have two factors?”, which earlier validations answered, to the question “How should the instrument be scored?”, which they did not address.

Compared with the earlier Polish validation by Studnicka et al. (25), which was limited to nursing students and used only exploratory factor analysis with maximum-likelihood estimation, the present study considerably extends the evidence base in three directions. First, our sample is larger and spans four healthcare disciplines, although it remains a single-center convenience sample in which nursing students predominate, so the extension concerns the range of disciplines represented rather than representativeness in the statistical sense. Second, we used CFA with WLSMV estimation, which is the current methodological standard for ordinal Likert items and produces more trustworthy parameter estimates than Pearson-based approaches (30, 31). Third, we applied advanced reliability and added-value indices, which provide a more nuanced picture of how the instrument should be used in practice. Our findings are fully compatible with the two-factor structure reported by Studnicka et al., but they temper the interpretation of subscale scores and call for item revision before the instrument is relied upon for decisions about individual learners.

For applied use, the two scoring options serve different purposes, and both remain defensible provided their limits are respected. The total score is the more precise estimate of a student’s overall appraisal of a simulation session and should be the default whenever the purpose is routine course evaluation, monitoring of a simulation center over time, or comparison of successive cohorts within the same programme: it is the option supported by the added-value analysis, and it carries the highest reliability of the three scores examined. The subscale scores remain useful when the purpose is diagnostic rather than summative. If a teaching team observes a disappointing overall rating, it matters a great deal whether the shortfall lies in satisfaction with the design, pacing, and debriefing of the scenario, or in students’ confidence in performing the clinical task itself, because those two situations call for different curricular responses–the first for redesign of the session, the second for additional deliberate practice and formative feedback. In such applications the subscale scores should be read as descriptive profiles at group level, not as separate individually interpretable measurements, and they should never be used to rank or compare individual students.

The ceiling effects observed across items (negative skewness, concentration of responses at the upper end of the scale) deserve attention. Such patterns are common in volunteer samples of satisfied or engaged learners and may be amplified by social-desirability bias in educational evaluations, notwithstanding the anonymity procedures used here. They suggest that the current response format (5-point Likert) may have limited capacity to discriminate among students with uniformly positive attitudes. Future revisions could explore wider response formats (e.g., 7-point Likert) or the addition of negatively worded items to reduce response-style effects and improve measurement precision. The modest marginal reliability reported above is the quantitative expression of the same phenomenon, and the two findings should be read together rather than as separate weaknesses.

The educational implications of these findings are concrete but deliberately bounded. Within institutions that resemble the one studied, the PL-SZZ offers a short, freely administrable instrument that can be embedded at the end of a simulation block to give teaching teams standardized feedback on how a scenario was received, to track change following a curricular modification, and to compare the reception of different scenario designs within the same programme. It does not measure competence, knowledge, or transfer to clinical practice, and it should be used alongside–never in place of–performance-based assessment. Because our data show no difference between simulation modalities but significant differences between fields of study, teaching teams using the instrument should interpret scores within a discipline rather than across disciplines until measurement invariance has been established.

Four lines of further research follow directly from the limitations of the present work. First, measurement invariance across fields of study, sex, and institutions should be tested in a multi-center sample designed prospectively with adequate subgroup sizes. Until this is done, between-group comparisons of PL-SZZ scores are not interpretable. Second, a test–retest administration over a short interval, during which no simulation teaching intervenes, would establish the temporal stability of the scores, which the present cross-sectional design could not address. Third, criterion and predictive validity should be examined against independent indicators of educational outcome–objective structured clinical examination results, rated debriefing quality, or subsequent clinical performance–none of which were available here. This is the most substantial evidential gap remaining for the Polish version. Fourth, item revision should be evaluated experimentally rather than assumed: a wider response format, or the addition of items targeting the upper region of the latent continuum, would test directly whether the ceiling effects and the modest factor-score precision observed in this study can be reduced.

### Strengths and limitations

The present study represents the first comprehensive evaluation of the Polish version of the Student Satisfaction and Self-Confidence in Learning scale (PL-SZZ) using confirmatory factor analysis with an estimator appropriate for ordinal data. In addition to evaluating the latent structure of the instrument, the study incorporated contemporary psychometric approaches, including marginal reliability, HTMT, and empirical added-value analysis, thereby providing a broader perspective on score interpretation than that afforded by traditional reliability coefficients alone. Furthermore, the translation and cultural adaptation followed established cross-cultural adaptation procedures, including expert review and back-translation, documented in five stages.

Several limitations should nevertheless be considered when interpreting the findings. First, although rigorous data-screening procedures were applied before the analyses, the exclusion of questionnaires during the quality-control process may have affected the representativeness of the final sample. While the retained sample remained sufficiently large for the planned analyses and included students from multiple healthcare disciplines, the possibility that excluded participants differed systematically from those included cannot be entirely excluded. Second, the study used a convenience sample recruited according to the teaching schedule of a single simulation center, and the representation of the four fields of study is markedly uneven: nursing students constitute nearly two-thirds of the sample, whereas Emergency Medical Services students constitute one twentieth. The resulting estimates are therefore weighted heavily toward the nursing experience of simulation, and the psychometric properties reported here should not be assumed to hold in disciplines that are sparsely represented in this dataset. Third, the number of questionnaires distributed was not recorded, so no institutional response rate can be reported, and the extent of any non-response bias cannot be quantified. Fourth, participants were recruited from a single academic institution using convenience sampling; consequently, the generalizability of the findings to students from other Polish universities and educational settings requires further confirmation. Fifth, the marked predominance of female participants (86.6%) reflects the demographic structure of many healthcare programmes in Poland but limits the precision of conclusions regarding male students, and in particular means that the instrument’s behavior in male-dominated or highly technical clinical training settings remains untested. Sixth, the cross-sectional design precluded the assessment of test–retest reliability, that is, of the temporal stability of the instrument, responsiveness to educational interventions, and other longitudinal aspects of validity. Seventh, although the correlated two-factor structure was supported, measurement invariance across disciplines, sex, or other relevant subgroups was not examined, for the reasons of statistical power set out in the section “Statistical analysis.” Consequently, the comparability of PL-SZZ scores across these groups should be established in future studies before the instrument is used for substantive between-group comparisons.

Three further limitations concern the evidence base for validity itself. First, no external criterion of educational outcome–such as examination performance, rated clinical competence, or independently assessed debriefing quality–was available in this design, so criterion and predictive validity of the PL-SZZ remain unevaluated. The validity evidence presented here is confined to internal structure and internal consistency. Second, content validity rests on structured expert review and back-translation, without quantified item-level and scale-level content validity indices and without a cognitive-debriefing round with students, so the cultural alignment of the Polish wording is supported but not formally quantified. Third, classical item-discrimination indices were not reported, because the restricted response variance in this sample renders them uninformative. Readers accustomed to seeing corrected item–total correlations in validation reports should note this omission and its rationale.

## Conclusion

The findings of the present study provide evidence supporting the reliability and construct validity of the Polish version of the Student Satisfaction and Self-Confidence in Learning scale (PL-SZZ) in a single-center, predominantly female and predominantly nursing sample of Polish healthcare students. The results are consistent with the original correlated two-factor conceptualization of the instrument, while the empirical added-value analysis suggests that the total score captures the information relevant to estimating the underlying latent dimensions at least as well as the corresponding subscale scores. These findings support the use of the PL-SZZ as a group-level measure of students’ perceptions of simulation-based learning in Polish healthcare education settings comparable to the one studied, with the total score as the default reporting unit and the subscale scores retained for diagnostic, curriculum-development purposes.

These conclusions apply to the population examined and should not be extended, without further evidence, to other institutions, to male-dominated or highly technical training settings, to comparisons between fields of study or between women and men, or to decisions concerning individual students. Nevertheless, further studies are needed to examine the generalizability of these findings across other educational settings, evaluate measurement invariance across relevant subgroups, establish criterion and predictive validity against independent indicators of educational outcome, and investigate the longitudinal measurement properties of the instrument, including test–retest reliability and responsiveness to educational interventions.

## Data Availability

The original contributions presented in this study are included in this article/Supplementary material, further inquiries can be directed to the corresponding author.
