# Supplementary material for: Polish translation and validation of the student satisfaction and self-confidence in learning scale among healthcare students at the Pomeranian Medical University in Szczecin: a single-center cross-sectional validation study
Source: Front Med (Lausanne). 2026 Aug 25;13:1913838. doi: 10.3389/fmed.2026.1913838 (PMC13563758; doi:10.3389/fmed.2026.1913838)
Supplement: Supplementary file 1 [file Table_1.docx]

**Supplementary Figures S1–S2**

Schematic path diagrams of the two competing confirmatory factor models tested in the validation of the PL-SZZ (N = 827). Standardized factor loadings (λ) from the WLSMV solution are shown next to each path. All loadings were statistically significant at p < 0.001.

**Figure S1. One-factor model**

All 13 items loading on a single latent construct representing overall subjective contentment with simulation-based learning.

**Figure S2. Two-factor correlated model**

Items loading on two correlated first-order factors (Satisfaction and Self-confidence). Inter-factor correlation r = 0.86.

*Note.* In both panels, rectangles represent observed (measured) items, ellipses or rounded boxes represent latent factors, and numbers next to arrows are standardized factor loadings (λ) obtained with the WLSMV estimator.
