# Supplementary material for: Polish translation and validation of the student satisfaction and self-confidence in learning scale among healthcare students at the Pomeranian Medical University in Szczecin: a single-center cross-sectional validation study
Source: Front Med (Lausanne). 2026 Aug 25;13:1913838. doi: 10.3389/fmed.2026.1913838 (PMC13563758; doi:10.3389/fmed.2026.1913838)
Supplement: Supplementary file 2 [file Table_2.docx]

**Supplementary File 1**

**Polish version of the Student Satisfaction and Self-Confidence in Learning scale (PL-SZZ)**

*The Polish-language version of the National League for Nursing (NLN) Student Satisfaction and Self-Confidence in Learning (SSCL) scale used in the present study is reproduced below. The questionnaire was developed by the National League for Nursing (© 2005) and translated into Polish under the title Satysfakcja studenta i pewność siebie w nauce (PL-SZZ). Written permission to translate and use the scale was obtained from the NLN in November 2021.*

**Badanie satysfakcji studenta z zajęć w CSM**

**Część I. Dane ogólne**

Wydział: □ Medycyny i Stomatologii	□ Nauk o Zdrowiu

Kierunek: □ Lekarski	□ Lekarsko-dentystyczny	□ Pielęgniarstwo	□ Położnictwo	□ Ratownictwo medyczne

Rok studiów: ………………

Forma studiów: □ Stacjonarne	□ Niestacjonarne

Przedmiot: ………………………………

Rodzaj symulacji: □ Wysoka wierność	□ Niska wierność	□ Pacjent symulowany / standaryzowany

Płeć: □ K	□ M

**Część II. Instrukcja**

Poniższa ankieta jest serią stwierdzeń dotyczących Twojego osobistego stosunku do przeprowadzanych symulacji. Każdy podpunkt stanowi oświadczenie o Twoim stopniu zadowolenia z nauki. Nie ma dobrych ani złych odpowiedzi. Prawdopodobnie z niektórymi stwierdzeniami będziesz się zgadzał/a, a z innymi nie.

Proszę wskaż swoje osobiste odczucia dotyczące każdego z poniższych stwierdzeń, zaznaczając liczby, które najlepiej opisują Twoją postawę lub przekonania. Proszę bądź prawdomówny/a. Wyraź swoją szczerą opinię, a nie taką, jaką uważasz, że dobrze byłoby wskazać. Ankieta jest całkowicie anonimowa. Wnioski z badania wyciągniemy na podstawie opinii większej grupy, a nie pojedynczych osób.

**Skala oceny:**

1 = ZDECYDOWANIE NIE ZGADZAM SIĘ ze stwierdzeniem

2 = NIE ZGADZAM SIĘ ze stwierdzeniem

3 = NIE JESTEM ZDECYDOWANY/A czy się zgadzam, czy nie ze stwierdzeniem

4 = ZGADZAM SIĘ ze stwierdzeniem

5 = ZDECYDOWANIE ZGADZAM SIĘ ze stwierdzeniem

**Część III. Pozycje skali**

| **No.** | **SATYSFAKCJA Z OBECNEGO SPOSOBU NAUKI** | **1** | **2** | **3** | **4** | **5** |
| --- | --- | --- | --- | --- | --- | --- |
| 1 | Zastosowane w symulacji sposoby nauczania były pomocne i efektywne. | □ | □ | □ | □ | □ |
| 2 | Symulacja stała się dobrą podstawą do realizacji programu przedmiotu. | □ | □ | □ | □ | □ |
| 3 | Podobał mi się sposób, w jaki asystent prowadził symulację. | □ | □ | □ | □ | □ |
| 4 | Materiały dydaktyczne zastosowane w symulacji były motywujące i pomogły mi lepiej zrozumieć omawiane zagadnienie. | □ | □ | □ | □ | □ |
| 5 | Sposób, w jaki nauczyciel prowadził symulację, był zbieżny z moim sposobem uczenia się. | □ | □ | □ | □ | □ |
| **No.** | **PEWNOŚĆ SIEBIE W NAUCE** | **1** | **2** | **3** | **4** | **5** |
| 6 | Jestem przekonany/na, że opanowałem/am ćwiczenia prezentowane podczas symulacji. | □ | □ | □ | □ | □ |
| 7 | Jestem przekonany/na, że symulacja obejmowała kluczowe treści niezbędne do opanowania programu nauczania przedmiotu. | □ | □ | □ | □ | □ |
| 8 | Jestem przekonany/na, że rozwinąłem/ęłam swoje umiejętności i zdobyłem/am wymaganą wiedzę, która przyczyni się do wykonania niezbędnych zadań w warunkach klinicznych / naturalnych. | □ | □ | □ | □ | □ |
| 9 | Nauczyciele byli przygotowani do realizacji symulacji i wykorzystali wszystkie możliwe pomoce. | □ | □ | □ | □ | □ |
| 10 | Moim obowiązkiem jako studenta/ki jest opanowanie efektów uczenia się wynikających z symulacji. | □ | □ | □ | □ | □ |
| 11 | Wiem, jak uzyskać pomoc, gdy nie rozumiem omawianych zagadnień w symulacji. | □ | □ | □ | □ | □ |
| 12 | Wiem, jak wykorzystać ćwiczenia symulacyjne do poznania kluczowych aspektów tych umiejętności. | □ | □ | □ | □ | □ |
| 13 | Obowiązkiem nauczyciela jest wyjaśnienie, które z omawianych zagadnień powinny zostać opanowane podczas symulacji. | □ | □ | □ | □ | □ |

*© Copyright, National League for Nursing, 2005. Polish translation: Bielikowicz et al. (2025).*
