# Supplementary material for: Polish translation and validation of the student satisfaction and self-confidence in learning scale among healthcare students at the Pomeranian Medical University in Szczecin: a single-center cross-sectional validation study
Source: Front Med (Lausanne). 2026 Aug 25;13:1913838. doi: 10.3389/fmed.2026.1913838 (PMC13563758; doi:10.3389/fmed.2026.1913838)
Supplement: Supplementary file 3 [file Table_3.docx]

**Supplementary File 2**

**Polish version of the Student Satisfaction and Self-Confidence in Learning scale (PL-SZZ)**

*The Polish-language version of the National League for Nursing (NLN) Student Satisfaction and Self-Confidence in Learning (SSCL) scale used in the present study is reproduced below. The questionnaire was developed by the National League for Nursing (© 2005) and translated into Polish under the title Satysfakcja studenta i pewność siebie w nauce (PL-SZZ). Written permission to translate and use the scale was obtained from the NLN in November 2021.*

**Part I. General Information**

**Faculty:** □ Medicine and Dentistry	□ Health Sciences

**Field of study:** □ Medicine	□ Dentistry	□ Nursing	□ Midwifery	□ Emergency Medical Services

**Year of study:** ……………………

**Mode of study:** □ Full-time	□ Part-time

**Subject:** ……………………………………………………

**Type of simulation:** □ High fidelity	□ Low fidelity	□ Standardized patient

**Sex:** □ F	□ M

**Part II. Instructions**

This questionnaire is a series of statements about your personal attitudes about the instruction you receive during your simulation activity. Each item represents a statement about your attitude toward your satisfaction with learning and self-confidence in obtaining the instruction you need. There are no right or wrong answers. You will probably agree with some of the statements and disagree with others.

Please indicate your own personal feelings about each statement below by marking the numbers that best describe your attitude or beliefs. Please be truthful and describe your attitude as it really is, not what you would like for it to be. This is anonymous with the results being compiled as a group, not individually.

**Rating scale:**

1 = STRONGLY DISAGREE with the statement

2 = DISAGREE with the statement

3 = UNDECIDED – you neither agree or disagree with the statement

4 = AGREE with the statement

5 = STRONGLY AGREE with the statement

**Part III. Scale Items**

| **No.** | **SATISFACTION WITH CURRENT LEARNING** | **1** | **2** | **3** | **4** | **5** |
| --- | --- | --- | --- | --- | --- | --- |
| 1 | The teaching methods used in this simulation were helpful  and effective. | □ | □ | □ | □ | □ |
| 2 | The simulation provided me with a variety of learning materials and activities to promote my learning the medical surgical curriculum. | □ | □ | □ | □ | □ |
| 3 | I enjoyed how my instructor taught the simulation. | □ | □ | □ | □ | □ |
| 4 | The teaching materials used in this simulation were motivating and helped me to learn. | □ | □ | □ | □ | □ |
| 5 | The way my instructor(s) taught the simulation was suitable  to the way I learn. | □ | □ | □ | □ | □ |
| **No.** | **SELF-CONFIDENCE IN LEARNING** | **1** | **2** | **3** | **4** | **5** |
| 6 | I am confident that I am mastering the content of the simulation activity that my instructors presented to me. | □ | □ | □ | □ | □ |
| 7 | I am confident that this simulation covered critical content necessary for the mastery of medical surgical curriculum. | □ | □ | □ | □ | □ |
| 8 | I am confident that I am developing the skills and obtaining  the required knowledge from this simulation to perform necessary tasks in a clinical setting. | □ | □ | □ | □ | □ |
| 9 | My instructors used helpful resources to teach the simulation. | □ | □ | □ | □ | □ |
| 10 | It is my responsibility as the student to learn what I need to know from this simulation activity. | □ | □ | □ | □ | □ |
| 11 | I know how to get help when I do not understand the concepts covered in the simulation. | □ | □ | □ | □ | □ |
| 12 | I know how to use simulation activities to learn critical aspects of these skills. | □ | □ | □ | □ | □ |
| 13 | It is the instructor’s responsibility to tell me what I need to learn of the simulation activity content during class time. | □ | □ | □ | □ | □ |

*© Copyright, National League for Nursing, 2005. Polish translation: Bielikowicz et al. (2025).*
