# Supplementary material for: Polish translation and validation of the student satisfaction and self-confidence in learning scale among healthcare students at the Pomeranian Medical University in Szczecin: a single-center cross-sectional validation study
Source: Front Med (Lausanne). 2026 Aug 25;13:1913838. doi: 10.3389/fmed.2026.1913838 (PMC13563758; doi:10.3389/fmed.2026.1913838)
Supplement: Supplementary file 4 [file Table_4.docx]

**Supplementary Table S1**

**Table S1.** Comparative analyses of PL-SZZ total and subscale scores by simulation modality, field of study, and sex (N = 827).

**Panel A. Score comparison between participants from different fields of study**

| **Variable** | **M (SD)** | | | | **H** | **ε^2^** | **post-hoc (Dunn)** |
| --- | --- | --- | --- | --- | --- | --- | --- |
|  | **Medicine ^A^**  **(N = 117)** | **Nursing ^B^**  **(N = 534)** | **Obstetrics ^C^**  **(N = 131)** | **EMS ^D^**  **(N = 45)** |  |  |  |
| Total score | 4.64 (0.32) | 4.66 (0.40) | 4.78 (0.34) | 4.66 (0.38) | **14.77 **** | .018 | A – B ^ns^  **A < C *****  A – D ^ns^  **B < C ***  B – D ^ns^  C – D ^ns^ |
| Satisfaction | 4.66 (0.41) | 4.66 (0.47) | 4.79 (0.38) | 4.72 (0.44) | **11.13 *** | .013 | A – B ^ns^  **A < C ****  A – D ^ns^  **B < C ****  B – D ^ns^  C – D ^ns^ |
| Self-confidence | 4.63 (0.34) | 4.66 (0.41) | 4.77 (0.36) | 4.63 (0.37) | **15.00 **** | .018 | A – B ^ns^  **A < C ****  A – D ^ns^  B – C ^ns^  B – D ^ns^  C – D ^ns^ |

Note. *M – mean, SD – std. deviation, H – Kruskal-Wallis ANOVA, ε2 – epsilon-squared effect size. All post-hoc comparisons were carried with use of the Dunn’s test with Bonferroni correction for multiple-comparisons. ns – non-significant, * - p < 0,05, ** - p < 0,01, *** - p < 0,001*

**Panel B. Score comparison between participants of different simulation types**

| **Variable** | **M (SD)** | | | **H** | **p** |
| --- | --- | --- | --- | --- | --- |
|  | **High-fidelity**  **(N = 473)** | **Low-fidelity**  **(N = 204)** | **Standardized patient**  **(N = 150)** |  |  |
| Total score | 4.67 (0.38) | 4.68 (0.42) | 4.68 (0.34) | 1.68 ^ns^ | .431 |
| Satisfaction | 4.68 (0.45) | 4.69 (0.47) | 4.66 (0.42) | 2.10 ^ns^ | .351 |
| Self-confidence | 4.67 (0.39) | 4.67 (0.43) | 4.69 (0.36) | 0.79 ^ns^ | .675 |

Note. *M – mean, SD – std. deviation, H – Kruskal-Wallis ANOVA. p – p-value. All comparisons were non-significant (ns)*

**Panel C. Score comparison between participants from different faculty**

| **Variable** | **M (SD)** | | **U** | **r^rb^** |
| --- | --- | --- | --- | --- |
|  | **Medicine and dentistry (N = 117)** | **Health Sciences**  **(N = 710)** |  |  |
| Total score | 4.62 (0.32) | 4.68 (0.39) | **35558.5 *** | -.15 |
| Satisfaction | 4.66 (0.41) | 4.68 (0.46) | 38192.0 ^ns^ | -.08 |
| Self-confidence | 4.63 (0.34) | 4.68 (0.40) | **35903.0 *** | -.14 |

Note. *M – mean, SD – std. deviation, U – Mann’s-Whitney U test, r^rb^ – rank-biserial correlation coefficient ns – non-significant, * - p < 0,05*

**Panel D. Score comparison between participants of different sex**

| **Variable** | **M (SD)** | | **U** | **r^rb^** |
| --- | --- | --- | --- | --- |
|  | **Female (N = 716)** | **Male**  **(N = 111)** |  |  |
| Total score | 4.66 (0.39) | 4.77 (0.33) | **33833.0 **** | -.15 |
| Satisfaction | 4.67 (0.46) | 4.78 (0.39) | **34292.5 *** | -.14 |
| Self-confidence | 4.66 (0.40) | 4.76 (0.33) | **34185.5 *** | -.14 |

Note. *M – mean, SD – std. deviation, U – Mann’s-Whitney U test, r^rb^ – rank-biserial correlation coefficient, * - p < 0,05, , ** - p < 0,01*
