# Supplementary material for: Polish translation and validation of the student satisfaction and self-confidence in learning scale among healthcare students at the Pomeranian Medical University in Szczecin: a single-center cross-sectional validation study
Source: Front Med (Lausanne). 2026 Aug 25;13:1913838. doi: 10.3389/fmed.2026.1913838 (PMC13563758; doi:10.3389/fmed.2026.1913838)
Supplement: Supplementary file 5 [file Table_5.docx]

**Supplementary Table S2**

**Table S2. Item- and scale-level descriptive statistics for the PL-SZZ (N = 827)**

| **Item / Scale** | **M (SD)** | **Min–Max** | **Skewness** | **Kurtosis** | **Shapiro–Wilk W** | **N (%) of responses** | | | | |
| --- | --- | --- | --- | --- | --- | --- | --- | --- | --- | --- |
|  |  |  |  |  |  | **1** | **2** | **3** | **4** | **5** |
| Item 1 | 4.68 (0.56) | 1 – 5 | -1.75 | 3.45 | 0.598 | 1 (0.1) | 1 (0.1) | 30 (3.6) | 200 (24.2) | 595 (71.9) |
| Item 2 | 4.71 (0.54) | 2 – 5 | -1.97 | 4.19 | 0.563 | - | 5 (0.6) | 21 (2.5) | 180 (21.8) | 621 (75.1) |
| Item 3 | 4.77 (0.48) | 3 – 5 | -1.98 | 3.17 | 0.514 | - | - | 22 (2.7) | 136 (17.6) | 659 (79.7) |
| Item 4 | 4.68 (0.56) | 2 – 5 | -1.61 | 2.08 | 0.601 | - | 2 (0.2) | 32 (3.9) | 197 (23.8) | 596 (72.1) |
| Item 5 | 4.56 (0.77) | 1 – 5 | -2.09 | 4.93 | 0.617 | 9 (1.1) | 9 (1.1) | 59 (7.1) | 180 (21.8) | 570 (68.9) |
| Item 6 | 4.42 (0.70) | 2 – 5 | -0.89 | -0.15 | 0.741 | - | 4 (0.5) | 90 (10.9) | 284 (34.3) | 449 (54.3) |
| Item 7 | 4.64 (0.56) | 2 – 5 | -1.36 | 1.34 | 0.634 | - | 2 (0.2) | 28 (3.4) | 235 (28.4) | 562 (68.0) |
| Item 8 | 4.59 (0.60) | 2 – 5 | -1.22 | 0.89 | 0.667 | - | 2 (0.2) | 43 (5.2) | 250 (30.2) | 532 (64.3) |
| Item 9 | 4.78 (0.46) | 2 – 5 | -2.15 | 4.93 | 0.503 | - | 2 (0.2) | 11 (1.3) | 151 (18.3) | 663 (80.2) |
| Item 10 | 4.75 (0.47) | 3 – 5 | -1.61 | 1.64 | 0.550 | - | - | 14 (1.7) | 181 (21.9) | 632 (76.4) |
| Item 11 | 0.42 (0.52) | 1 – 5 | -1.95 | 4.79 | 0.563 | 1 (0.1) | 1 (0.1) | 20 (2.4) | 185 (22.4) | 620 (75.0) |
| Item 12 | 4.66 (0.55) | 2 – 5 | -1.39 | 1.21 | 0.623 | - | 1 (0.1) | 30 (3.6) | 222 (26.8) | 574 (69.4) |
| Item 13 | 4.80 (0.42) | 3 – 5 | -1.88 | 2.47 | 0.489 | - | - | 7 (0.8) | 148 (17.9) | 672 (81.3) |
| **Satisfaction subscale** | 4.68 (0.45) | 3.40 – 5.00 | -1.16 | -0.01 | 0.73 |  |  |  |  |  |
| **Self-confidence subscale** | 4.67 (0.39) | 3.63 – 5.00 | -0.93 | -0.33 | 0.81 |  |  |  |  |  |
| **Total scale** | 4.67 (0.38) | 3.62 – 5.00 | -0.94 | -0.36 | 0.82 |  |  |  |  |  |

*Note.* M = mean; SD = standard deviation; Min–Max = observed range. Kurtosis is reported as excess kurtosis. All Shapiro–Wilk normality tests were significant at
p < 0.001, confirming the non-normal distribution of items typical of ordinal Likert data and justifying the use of the WLSMV estimator in confirmatory factor analysis. Multivariate normality tests (Mardia's skewness M-Sk = 69.76, p < 0.001; Mardia's kurtosis M-Ku = 412.25, p < 0.001) similarly indicated departures from multivariate normality.
