# Supplementary material for: Polish translation and validation of the student satisfaction and self-confidence in learning scale among healthcare students at the Pomeranian Medical University in Szczecin: a single-center cross-sectional validation study
Source: Front Med (Lausanne). 2026 Aug 25;13:1913838. doi: 10.3389/fmed.2026.1913838 (PMC13563758; doi:10.3389/fmed.2026.1913838)
Supplement: Supplementary file 6 [file Table_6.docx]

**Supplementary Table S3**

**Table S3. Extended goodness-of-fit evaluation of both confirmatory factor models with equivalence-testing (Yuan et al., 2016) and tailored (Grosskurth et al., 2023) cut-offs**

**Panel A. Absolute and relative fit indices with tailored cut-offs (Grosskurth et al., 2023)**

| **Index** | **1-factor** | **2-factor** |
| --- | --- | --- |
| χ² | 642.69 | 401.89 |
| χ² tailored cut-off | 78.4 | 80.8 |
| Difference from cut-off | 564.3 (-) | 321.1 (-) |
| df | 103 | 101 |
| χ²/df | 6.240 | 3.979 |
| χ²/df tailored cut-off | 1.195 | 1.212 |
| Difference from cut-off | 5.045 (-) | 2.767 (-) |
| CFI | 0.949 | 0.978 |
| CFI tailored cut-off | 0.989 | 0.978 |
| Difference from cut-off | 0.040 (-) | 0.000 |
| RMSEA | 0.080 | 0.060 |
| RMSEA 90% CI | 0.074 – 0.086 | 0.033 – 0.057 |
| RMSEA tailored cut-off | 0.014 | 0.013 |
| Difference from cut-off | 0.066 (-) | 0.047 (-) |
| SRMR | 0.066 | 0.050 |
| SRMR tailored cut-off | 0.008 | 0.018 |
| Difference from cut-off | 0.058 (-) | 0.032 (-) |

**Panel B. Equivalence-testing cut-offs (Yuan et al., 2016) for non-centrality-based indices**

| **Index** | **1-factor** | **2-factor** |
| --- | --- | --- |
| RMSEA T-size | 0.086 | 0.066 |
| Poor-fair limit | 0.087 | |
| Fair-close limit | 0.056 | |
| Diff. from P-F limit | 0.001 (+) | 0.021 (+) |
| Diff. from F-C limit | 0.030 (-) | 0.010 (-) |
| CFI T-size | 0.970 | 0.982 |
| Poor-fair limit | 0.930 | |
| Fair-close limit | 0.870 | |
| Diff. from P-F limit | 0.040 (+) | 0.052 (+) |
| Diff. from F-C limit | 0.100 (+) | 0.112 (+) |

*Note.* F-C = fair-close fit limit; P-F = poor-fair fit limit; T-size = minimum tolerable size of misspecification at the 95% confidence level (Yuan et al., 2016). Tailored cut-offs are derived from regression formulae proposed by Grosskurth et al., 2023 based on their simulation studies. (+) indicates better fit than proposed by cut-off, (-) indicate worse fit.
Both models didn’t satisfy the close-fit criteria for χ²/, χ²/df, CFI, RMSEA and SRMR under approach proposed by Grosskurth et al., but CFI and RMSEA shown at least fair fit under equivalence-testing approach proposed by Yuan et al.
