# Supplementary material for: Polish translation and validation of the student satisfaction and self-confidence in learning scale among healthcare students at the Pomeranian Medical University in Szczecin: a single-center cross-sectional validation study
Source: Front Med (Lausanne). 2026 Aug 25;13:1913838. doi: 10.3389/fmed.2026.1913838 (PMC13563758; doi:10.3389/fmed.2026.1913838)
Supplement: Supplementary file 7 [file Table_7.docx]

**Supplementary Table S4**

**Table S4. Standardized factor loadings for the two competing CFA models (N = 827)**

| **Factor** | **Item** | **1-factor model (λ)** | **2-factor model (λ)** |
| --- | --- | --- | --- |
| *Satisfaction* | Item 1 | 0.86 | 0.89 |
|  | Item 2 | 0.84 | 0.86 |
|  | Item 3 | 0.84 | 0.87 |
|  | Item 4 | 0.85 | 0.88 |
|  | Item 5 | 0.72 | 0.75 |
| *Self-confidence* | Item 6 | 0.82 | 0.84 |
|  | Item 7 | 0.85 | 0.87 |
|  | Item 8 | 0.82 | 0.84 |
|  | Item 9 | 0.80 | 0.82 |
|  | Item 10 | 0.74 | 0.75 |
|  | Item 11 | 0.78 | 0.80 |
|  | Item 12 | 0.83 | 0.85 |
|  | Item 13 | 0.69 | 0.70 |
| **Mean λ (overall)** |  | 0.80 | 0.83 |
| **Mean λ – Satisfaction** |  | 0.82 | 0.85 |
| **Mean λ – Self-confidence** |  | 0.79 | 0.81 |

*Note.* All factor loadings are standardized and were statistically significant at p < 0.001. λ = standardized factor loading.
Loadings for all items were slightly higher in two-factor model.
